# Self-regulated learning in a competency-based and flipped learning environment: learning strategies across achievement levels and years

**DOI:** 10.1080/10872981.2019.1686949

**Published:** 2019-11-01

**Authors:** Binbin Zheng, Amy Ward, Randi Stanulis

**Affiliations:** Office of Medical Education Research and Development, Michigan State University, East Lansing, MI, USA

**Keywords:** Self-regulated learning, learning strategies, flipped classroom, competency-based curriculum

## Abstract

**Background**: The transition from a traditional lecture-based curriculum to a competency-based curriculum poses significant challenges to both students and faculty in medical schools, especially when the curriculum is implemented in a flipped learning environment. Self-regulated learning (SRL) has been proven to be beneficial for competency-based learning and flipped classroom learning, but medical educators cannot expect our entering medical students to bring in these learning skills automatically.

**Methods**: This study took place in the Michigan State University College of Human Medicine. A new competency-based curriculum was implemented in the fall 2016, focusing on the integration of basic science and clinical experience. Participants in this study were 26 first- and second-year students. By interviewing each student about the learning strategies they use in independent learning before class, we investigated how students use SRL strategies in different phases of learning, and how their adoption of SRL strategies differ across self-perceived achievement groups and years.

**Results**: We found that students frequently use strategies in the stages of planning and reflection, but less frequently during the learning or monitoring phase. Students who perceive themselves as high achieving, and students in their second year of medical school do use more learning strategies during the monitoring stage than their counterparts.

**Conclusions**: Students who lack self-regulation strategies may fail to comprehend or connect ideas in their pre-class learning, which could lead to ineffective learning outcomes during in-class activities. Our study indicated that while medical students, who tend to be successful learners in their undergraduate study, were able to use learning strategies to plan and reflect on their learning, they need more explicit instruction in how to monitor their own learning.

## Introduction

New approaches in medical education prepare students to develop professional competencies that match with the knowledge and skills needed to become effective physicians [1]. These new approaches regularly promote small-group problem solving and critical thinking competencies in discussions and simulations. The transition from a traditional lecture-based curriculum to a competency-based curriculum poses significant challenges to both students and faculty in medical schools, especially when the curriculum is implemented in a flipped learning environment [2]. As part of our competency-base curriculum, each week students review 15 hours of online readings and videos in a flipped learning format in order to prepare for classroom-based activities. However, faculty rarely teach students how to conduct independent learning, assuming that students come to medical school with explicit strategies for learning voluminous material on their own.

Self-regulated learning (SRL) is viewed as ‘proactive processes that students use to acquire academic skill’ [3, p.166], including the cognitive, metacognitive, motivational, behavioral, and emotional aspects of learning [4]. It is a constructive process during which learners set up goals, and attempt to monitor, regulate, and control their cognition, motivation, and behavior [5]. Researchers suggest that SRL is critical in students’ learning success in face-to-face education [3]. For example, in a meta-analysis [6] the authors reported that students’ cognitive and meta-cognitive strategies both had positive impacts on student learning outcomes, with effect sizes of 0.60 and 0.53 respectively. As such, SRL skills, such as planning and self-monitoring one’s learning behaviors, have been considered an essential competency for students to attain their academic goals.

Self-regulation is an important factor for learning success in flipped classrooms, as students who lack self-regulation may learn little or fail to comprehend content before class, which will further lead to less effective learning outcomes or disengagement in subsequent in-class activities [7]. On the contrary, students with better self-regulation would be able to more effectively utilize and learn materials [8]. Thus, developing self-regulated learning strategies is essential for students to perform well in flipped learning environment.

Research on SRL is gaining momentum in medical education, emphasizing the importance of engaging medical students in effective self-regulated learning to better prepare them as future physicians [9,10] and to ensure the quality of patient care [11]. SRL has proven to be beneficial for competency-based learning and flipped classroom learning [7,12]. The purpose of this study was to identify the SRL strategies medical students use in a flipped learning environment, and further examine whether students with different levels of self-perceived academic performance use SRL differently., this study is guided by three research questions:

1. What SRL strategies did medical students use to prepare for their weekly learning in a flipped classroom model?
2. How did students use SRL strategies across different self-perceived achievement groups?
3. How did students use SRL strategies across different years in medical school?

## Methods

A new competency-based curriculum was implemented at Michigan State University in the fall 2016, focusing on the integration of basic science and clinical experience. In order to understand how students use SRL strategies in independent learning before class in this new curriculum, we adopted a qualitative research approach [13]. The study was determined to be exempt by the Institutional Review Board (IRB) at Michigan State University (MSU). The second author invited faculty to recommend students across achievement levels to be interviewed, including high achieving, struggling, and students who made a jump in achievement across the year. The 20 nominated students were invited to participate in a voluntary, confidential interview and were provided a $10 gift card for their participation. While a few students were unable to participate due to scheduling, 16 out of 20 students participated. The second author conducted all of the 20 minutes interviews using a semi-structured interview protocol. During the interview, students were asked to explain their process for studying for a typical week. After collecting basic demographic information, including the student’s self-perceived strength as a medical student, the interview began with the prompt, ‘Imagine it is Friday afternoon and you are about to start a new week. Describe what you do first, and then talk through your study process for the week providing as much detail as possible.’ The interviewer asked follow-up questions as needed to better understand what students did while studying.

Once we coded the 16 interviews and presented our initial results, the academic affairs leadership team asked us to open the interview process to more students, to see whether the patterns we found would remain consistent or change with a larger sample size. We opened up interview invitations to all first- and second-year students, accepting the first ten students who applied into our sample to see whether new themes emerged, and these students also received a $10 gift card each for their participation. After coding the additional 10 interviews, patterns were repeating and no new themes emerged.

All interviews were audio taped and transcribed. To analyze the interview data, we used focused coding chosen beforehand [14], based on our modification of a self-regulated learning framework [15]. All three researchers independently read interview responses to identify specific strategies students used within each phase of the SRL model, and met repeatedly to discuss emerging themes. The planning stage in SRL refers to preparing for learning by engaging in task analysis to determine what, how, and when material will be learned and connecting material strategically to prior knowledge to prepare to create frameworks for understanding [4]. Monitoring is the process of actively learning the material by creating frameworks to build understanding [4]. For this study, we grouped monitoring tasks into organizing, summarizing and applying – all of which are ways of adding meaning to new content. In SRL, reflection is the process of self-assessment that involves determining what information has been learned and adjusting for future learning based on that feedback [4].

As a result of data analysis, seven specific learning strategies in three constructive phases were identified and used as the coding scheme to analyze student interview responses: two in the planning phase before learning – task analysis and connecting; three in the monitoring phase during learning – summarizing, organizing, and applying; two in the reflecting stage after learning – self-evaluation and adjustment. See Table 1 for an overview of the framework and examples of each strategy.

**Table 1.** Self-regulated learning strategies framework (adopted and modified from Pintrich, 2004)

Phase	Learning strategy	Examples from our study
Planning	Task Analysis	Start by looking at objectives and resources
	Connecting	Use outside resources to understand readings
Monitoring	Organizing	Highlight important content and use one piece of paper with colors, images and connections to keep track of central ideas and make connections.
	Summarizing	Organize brief summary notes all on one sheet for week’s objectives.
	Applying	Put learning content in a particular clinical context
Reflecting	Self-assessment	Use objectives to create own quiz. If a student can answer the objective, stop there. If not, I would move on to the next reading that covers the same objective to achieve understanding.
	Adjustment	Moving from skim-reading everything to careful selective reading based on objectives.

For data analysis, descriptive statistics were used to describe the self-regulated learning strategies students use overall as well as across different levels of self-perceived performance and between first- and second-year students. Students self-identified their performance level as either low-, middle-, or high-achieving. Content analysis of students’ interviews were conducted using a focused coding strategy to categorize emerging themes about students’ specific strategy use.

## Results

A total of twenty-six students were interviewed. Ten were first year students and 16 were second year students. Among them, eight self-identified as low-achieving, 11 self-identified as mid-achieving, and seven self-identified as high-achieving students.

### Students’ overall use of SRL strategies

Using the SRL framework, we first analyzed all 26 students’ responses to the interview questions regarding their independent learning process when approaching their pre-class online learning modules. Our analysis revealed that overall, the most frequently used learning strategy was adjustment (73%), followed by task analysis (62%), connecting (31%), and self-evaluation (19%). These learning strategies fall under the phases of planning and reflection. In contrast, learning strategies during the monitoring phase such as organizing (11%), summarizing (8%), and applying (4%) were least frequently reported by our students.

### Planning

A total of twenty-four students (92%) engaged in planning activities.

#### Task analysis

More than half of the students (N = 16) reported analyzing the learning tasks first to strategically plan their pre-class learning. For task analysis, students described reading the objectives to gauge their workload, creating a checklist based on the objectives, or a combination. Some students analyzed the learning task more critically. One student mentioned that before he began reading, he would spend a few minutes pairing up the readings and objectives for the week to see how well the readings answered the objectives; because of this planning, he stated that he chose to read only when the readings answered an objective. Similarly, another student described that she decided what to read based on her understanding of the structure of the text – this meant that she planned what she would read by reading only the headings and diagrams of a text first and then deciding what material she would further delve into more extensively.

#### Connecting

Compared to task analysis, fewer students (N = 8) reported using connecting during the planning stage. Connecting refers to both making connections to the student’s own background knowledge and making connections among different resources. For example, some students mentioned that during planning they thought about previous notes and pulled those out in order to add to them. Others said that they liked to ‘ground’ themselves in background knowledge associated with what they were about to learn by watching an outside video or re-reading a resource.

### Monitoring

Only 6 (23%) of the students interviewed engaged in monitoring strategies. The most frequently used cognitive monitoring strategy, as reported by students, was organizing, followed by summarizing and applying. Far fewer students used monitoring (N = 6) than planning (N = 24) and reflecting (N = 24) strategies.

#### Organizing

The students (N = 3) who mentioned using an organizational monitoring strategy used charts, diagrams and tables to organize notes. One student described creating a chart with the central theme she was learning and then writing additional connected ideas around that theme.

#### Summarizing

Only two (N = 2) students mentioned the use of summarizing. In both cases the students talked about reading a section of text and then summarizing their learning to consolidate their knowledge and highlight what was important.

#### Applying

Only one (N = 1) student mentioned the use of applying. The student who mentioned the use of an applying strategy noted that during learning, he would try to put everything in the context of the disease process. By taking the content he was learning, and placing it into the larger context of a disease, he integrated his knowledge and thinking about how the science and clinical aspects of his learning overlapped.

### Reflecting

Like planning, most students (N = 24; 92%) reportedly engaged in some aspects of reflection. Five students mentioned the use of self-assessment and 19 students mentioned the use of adjustment during reflection.

#### Self-assessment

Students who described using self-assessment (N = 5) talked about using the stated learning objectives to quiz themselves. One student said that if he could not answer the objective, he would return to the list of readings and either re-read an article or read an article he hadn’t read yet.

#### Adjustment

Overall, 19 students talked about how they engaged in adjustment. Students explained they adjusted their learning behavior when they realized their traditional way of passively reading did not work in the new curriculum. While many of these students did not describe specific self-assessment strategies, they did engage in some form of adjustment based on broader feedback about their learning. Students mentioned that they started the year by reading everything, and then realized that that strategy did not work because it was either very time consuming, or they weren’t retaining the information. Some of the students described that they adjusted by reading selectively based on the learning objectives, while others described adding in outside resources to learn the material because those audio or video resources fit better to their own learning style.

### Students’ use of SRL strategies across self-perceived achievement

Among the 26 interviewees, 8 students identified themselves as low-achieving students, 11 identified themselves as middle-achieving students, and 7 identified themselves as high-achieving students. While comparing students’ use of SRL across their self-perceived level of achievement, our results showed that students at all levels used planning and reflection strategies more than they used monitoring strategies. While middle-achieving students used learning strategies during their planning (N = 11) and reflection (N = 11) phases the most, low- and high-achieving students did not differ much in their strategy use during planning (N = 6 for low-achieving and N = 7 for high-achieving) and reflection (N = 7 for low-achieving and N = 6 for high-achieving) phases (see Table 2 for detailed numbers of strategy use among three achievement groups). Learning strategies during the monitoring phase were least frequently used across all achievement groups, but high-achieving students (N = 4; 57%) used these strategies more often than both low (N = 1; 12.5%) and middle-achieving (N = 1; 9%) students.

**Table 2.** Students reporting of self-regulated learning strategies across self-perceived achievement groups

		Planning		Monitoring			Reflection	
	Total Students	Task analysis	Connecting	Organizing	Summarizing	Applying	Self-assessment	Adjustment
Low-achieving	8	3	3	0	1	0	2	5
Mid-achieving	11	7	4	1	0	0	2	9
High-achieving	7	6	1	2	1	1	1	5
Total	26	16	8	3	2	1	5	19

### Students’ use of SRL strategies between years

Ten first-year and 16 second-year students participated in the interview. Table 3 shows the detailed numbers of strategy use between Year 1 and Year 2 students. Comparing their use of SRL strategies, more Year 2 students (N = 16) reported using learning strategies during planning – especially for task analysis – than Year 1 students (N = 8). While more Year 2 students reported using learning strategies during the monitoring stage than Year 1 students (N = 5 for Year 2 and N = 1 for Year 1), in both groups the number of students engaged in monitoring strategies was less than a third of the total number of students. First- and second-year students did not differ much in their use of learning strategies during the reflection stage (N = 10 for Year 1 and N = 14 for Year 2).

**Table 3.** Students reporting of self-regulated learning strategies between Year 1 and Year 2

		Planning		Monitoring			Reflection	
	Total Students	Task analysis	Connecting	Organizing	Summarizing	Applying	Self-assessment	Adjustment
Year 1 students	10	5	3	0	1	0	1	9
Year 2 students	16	11	5	3	1	1	4	10
Total	26	16	8	3	2	1	5	19

## Discussion

### Critical role of monitoring strategies for learning success

Targeted pre-class preparation is extremely important for students’ overall learning in a flipped class model [16], since students who lack self-regulation may fail to comprehend or connect ideas. This lack of SRL strategy use can lead to less effective learning outcomes during in-class activities. Therefore, the purpose of this study was to describe the SRL strategies medical students use in a flipped environment, and how their use of SRL strategies differ across students of different self-perceived achievement groups and students in different years of medical school. We found that most students used adjusting and planning in their learning, while few students used monitoring strategies, regardless of year in medical school, or self-perceived achievement. The disparate use of learning strategies between planning/reflecting and monitoring may be due to the addition of a flipped classroom model. Most of our medical students, like medical students everywhere, were academically successful in their traditional lecture-based k-16 learning environment. They arrive in medical school having already developed certain effective learning strategies. However, lecture-based or non-flipped curriculum usually does not require monitoring strategies since it is usually the instructors’ responsibility to monitor student learning through testing. In a traditional classroom, instructors introduce material, and through lecture and activities, create frameworks for students to use better understand the material. In a flipped-classroom model, students must self-monitor their learning and come in having already developed frameworks and basic understanding, ready to apply the learning to novel situations [6]. In our new curriculum, which heavily relies on students’ self-directed learning, monitoring strategies become increasingly important yet not commonly mastered by most students.

When looking into specific SRL strategies, previous research has emphasized the key role of self-monitoring [17,18]. Relatedly in SRL, Harris [17] suggested that without specific instruction of self-monitoring, students would not improve acquisition. Our study re-enforces this claim. Thus, it is important for curriculum developers and medical education faculty to help students develop monitoring strategies to be academically successful in flipped learning environment.

Although our results indicated that most students, even in their second year, do not engage in many monitoring strategies, we in no way underestimate the importance of the learning strategies students should be taught to use in the other two phases of learning. In fact, these three phases – planning, monitoring, and reflecting – are not isolated from one another and should be integrated. While it is undoubtedly important to teach students specific learning strategies, it is even more important to help them strategically apply an appropriate strategy in different contexts. This life-long learning skill is of critical importance for better preparing our medical students to be high quality physicians [7,11,19].

### Supporting students in developing monitoring strategies

Our study indicates that as students transition from a traditional classroom to the flipped learning environment, we cannot expect students to be equipped with necessary monitoring strategies. Researchers suggests that these strategies can be explicitly taught with opportunities for repeated practice [6], and that educators and curriculum developers have a role to play in scaffolding students’ development of such skills [9,20].

Scholars have proposed a model of co-regulated learning to help students develop SRL strategies [3]. Compared with self-regulated learning that relies completely on learner’s own cognitive and meta-cognitive processes, co-regulated learning emphasizes a shared learning experience that takes place between a learner and a more experienced person, and this more experienced person could be a trained coach, or even a near peer. Through co-regulation, the expert shares the cognitive and metacognitive thinking load with the learner to help develop learners’ self-regulated learning skills. Using co-regulation as a model, an instructor or curriculum developer could leverage their expertise to scaffold students learning of monitoring strategies. Below are a few potential prompts that educators might use to aid students in the development of monitoring strategies:

- What is the relationship between this content and other content?
- How does this content change the way you approach or think about previous content?
- What do you know about this content beyond what is written here?
- How can you visually organize these concepts into a chart, graph, diagram? How does that visual organization change your conception of the material?

Curriculum developers can also provide scaffolding for students to learn monitoring strategies by providing multimodal representations such as a concept map, flow chart, or graphic organizer. These can be integrated into learning activities to help students develop connections between concepts and model methods that students might use to monitor their own learning.

## Limitations and future directions

Our study focused on 26 medical students in a medical school transitioning from a traditional lecture-based curriculum to a new competency-based curriculum. With a limited sample size of students studied in our particular curriculum it is possible that our findings may not be generalizable to other learning contexts.

## Significance

A significant gap exists between students’ learning strategies developed in traditional learning and what are needed in a flipped classroom model [21], especially in the monitoring phase of SRL. Without engaging in cognitive monitoring as they move through their independent work, students struggle to integrate and synthesize information. Medical educators continue to ask about the nature of specific guidance that supports students in discovery learning [22]. When the learning structure changes, new learning strategies must be explicitly taught and practiced for students to be successful.

While most of current literature in SRL and medical education acknowledges the importance of SRL for student learning or focuses on examining the use of SRL strategies, few studies have looked into how the strategies differ across different student populations, especially in the context of competency-based curriculum and a flipped learning environment. In addition, more action plans for learner, teacher, and the learning environment are called for to help develop master learners [23]. Panadero [4], in his review of SRL models, also called for more fine-grained research on how specific strategies in SRL work. Our study fills the gap of current literature in SRL and medical education by not only examining medical students’ SRL strategies during planning, monitoring, and reflection stages of learning across achievement level and years, but also calling for explicit instruction from both clinician educators and curriculum developers to provide a roadmap for developing self-regulated learners.
